# 120 Using Implementation Science to Develop a TL1 D&I Science Training Implementation Plan – ADDENDUM

**DOI:** 10.1017/cts.2023.570

**Published:** 2023-06-14

**Authors:** Denise H. Daudelin, Alyssa Cabrera, Anna L. Thompson, Thomas W. Concannon, Robert Sege, Elizabeth Leary, Angie Mae Rodday

The above abstract [1] published without listing Denise H. Daudelin as an author.

The original abstract has been corrected online to rectify this omission.
